# A comparison of four technologies for detecting p53 aggregates in ovarian cancer

**DOI:** 10.3389/fonc.2022.976725

**Published:** 2022-09-08

**Authors:** Nicole Heinzl, Katarzyna Koziel, Elisabeth Maritschnegg, Astrid Berger, Elisabeth Pechriggl, Heidi Fiegl, Alain G. Zeimet, Christian Marth, Robert Zeillinger, Nicole Concin

**Affiliations:** ^1^ Molecular Oncology Group, Department of Obstetrics and Gynecology, Comprehensive Cancer Center-Gynecologic Cancer Unit, Medical University of Vienna, Vienna, Austria; ^2^ Department of Gynecology and Obstetrics, Innsbruck Medical University, Innsbruck, Austria; ^3^ Institute for Clinical and Functional Anatomy, Innsbruck Medical University, Innsbruck, Austria

**Keywords:** p53, protein aggregation, ovarian cancer, immunofluorescence staining, immunoprecipitation, ELISA, proximity ligation assay

## Abstract

The tumor suppressor protein p53 is mutated in half of all cancers and has been described to form amyloid-like structures, commonly known from key proteins in neurodegenerative diseases. Still, the clinical relevance of p53 aggregates remains largely unknown, which may be due to the lack of sensitive and specific detection methods. The aim of the present study was to compare the suitability of four different methodologies to specifically detect p53 aggregates: co-immunofluorescence (co-IF), proximity ligation assay (PLA), co-immunoprecipitation (co-IP), and the p53-Seprion-ELISA in cancer cell lines and epithelial ovarian cancer tissue samples. In 7 out of 10 (70%) cell lines, all applied techniques showed concordance. For the analysis of the tissue samples co-IF, co-IP, and p53-Seprion-ELISA were compared, resulting in 100% concordance in 23 out of 30 (76.7%) tissue samples. However, Co-IF lacked specificity as there were samples, which did not show p53 staining but abundant staining of amyloid proteins, highlighting that this method demonstrates that proteins share the same subcellular space, but does not specifically detect p53 aggregates. Overall, the PLA and the p53-Seprion-ELISA are the only two methods that allow the quantitative measurement of p53 aggregates. On the one hand, the PLA represents the ideal method for p53 aggregate detection in FFPE tissue, which is the gold-standard preservation method of clinical samples. On the other hand, when fresh-frozen tissue is available the p53-Seprion-ELISA should be preferred because of the shorter turnaround time and the possibility for high-throughput analysis. These methods may add to the understanding of amyloid-like p53 in cancer and could help stratify patients in future clinical trials targeting p53 aggregation.

## Introduction

Protein misfolding, aggregation, and amyloid formation have been associated with neurodegenerative diseases such as transmissible spongiform encephalopathies (TSEs), Alzheimer’s disease, Parkinson’s disease, and even diabetes type 2. The disease-causing agents of TSEs, such as Creutzfeldt-Jakob disease or bovine spongiform encephalopathy (BSE), are prions. They are a subclass of amyloid proteins with the unique characteristic of being infectious. Amyloids are aggregated proteins with enriched β-sheet structures running perpendicular to the fibril axis, resulting in a fibrillar structure (1). Misfolded forms of key proteins in neurodegenerative diseases, such as β-amyloid or tau in Alzheimer’s disease and α-synuclein in Parkinson’s disease, share some of the characteristics of prions and have therefore been named prionoids or prion-like proteins (2).

Intriguingly, the p53 protein has shown amyloid-like behavior, thereby, adding cancer to the class of protein aggregation diseases. A so-called aggregation-prone sequence has been identified in the hydrophobic core of the DNA-binding domain (3, 4). This region gets exposed upon conformational changes caused by mutation, leading to the formation of amyloid-like protein aggregates (4). The amyloid-like structures formed by mutant p53 protein have been detected in the cytoplasm and the nucleus in different types of cancer cell lines and tumors (3, 5–8). It has been proposed that misfolded mutant p53 protein exhibits an amyloid-like behavior by converting correctly folded wild-type p53 to a misfolded amyloid conformation (9). Moreover, mutant p53 can cause co-aggregation not only of wild-type p53 but also of other members of the p53 protein family, namely p63 and p73. Therefore, the amyloid-like behavior of p53 can provide a mechanistic explanation for the dominant-negative and gain-of-function (GOF) effects of p53 (3, 10). Recently, it has been shown that amyloid p53 exerts another characteristic feature of prions, which is cell-to-cell transmission leading to the induction of amyloid formation in neighboring cells (8).

*TP53* mutations are found in more than 50% of cancer cases. The mutation rate varies across different cancer types, including ovarian cancer (OC) in which *TP53* mutations are the most frequent genetic alteration and the hallmark of precancerous lesions. The most dominant OC subtype, high-grade serous ovarian cancer (HGSOC), is characterized by an almost ubiquitously presence of *TP53* mutations (11). The ability of p53 to form amyloid-like structures has been observed in HGSOC cells exhibiting cancer stem cell properties, where it is associated with chemoresistance (12, 13). This finding attracts attention to amyloid-like p53 as a new potential therapeutic target. The first inhibitor targeting aggregated p53, ReACp53, was shown to diminish p53 amyloid formation and rescue the p53 function *in vitro* and in pre-clinical testing *in vivo* (4). Further, the combination of carboplatin and ReACp53 enhanced tumor cell targeting in OC cancer cell lines and patient-derived HGSOC organoids (14).

Robust, sensitive, and reproducible methods to detect and characterize amyloid p53 are a prerequisite for future studies unraveling their clinical relevance and possible therapeutic intervention.

Currently, the state-of-the-art method for the detection of p53 aggregates is the immunofluorescence co-localization assay (co-IF) based on the co-localization of p53 and amyloid structures (5–7, 10, 12, 15–18). For the detection of those amyloid structures, various antibodies or amyloid-specific dyes are available, including Thioflavin T, Congo Red, the anti-oligomer antibody A11, and the anti-amyloid fibrils antibody OC. Both amyloid-specific dyes bind amyloid fibrils with β-sheet-rich structures, but their specificity remains limited. The A11 antibody detects prefibrillar oligomers, which are immunologically distinct from the fibrillar oligomers that are recognized by the OC antibody. Neither one detects natively folded proteins and monomers (19). However, co-staining of two epitopes in the same subcellular compartment does not prove that both epitopes are present within one molecule and therefore, co-IF does not allow specific detection of p53 aggregates. Co-immunoprecipitation using A11 or OC antibodies for the pull-down of amyloid protein followed by immunoblotting to analyze the p53 levels in the amyloid fractions showed that p53 is present as amyloid aggregates (16, 20).

In an earlier study, we developed a highly-sensitive ELISA-based assay, the p53-Seprion-ELISA, for the detection of p53 aggregates in cancer cell lines and fresh-frozen tissue (21). This assay is based on a high-molecular-weight polymeric ligand, selectively binding aggregated proteins including amyloid oligomers, proto-fibrils, and fibrils (22). The Seprion ligand was previously used to isolate and quantify aggregated forms of prion protein (23). By combining the Seprion ligand with an anti-p53 antibody, the ELISA specifically detects high-molecular-weight p53, but neither monomers, naturally occurring tetramers, or octamers. The most recent method for detecting p53 aggregates is the proximity ligation assay (PLA). This technique is based on two primary antibodies, which are bound by oligonucleotide-labeled proximity probes that form a DNA circle when bound in close proximity. The DNA circle serves as a template for the rolling-circle amplification (RCA) and the amplified DNA is detected by fluorescently labeled detection probes (24). The resulting distinct fluorescent spots can be quantified *via* microscopy or flow cytometry. The PLA has been successfully applied to detect oligomeric p53 aggregates in nuclear inclusion bodies in ovarian cancer tissue biopsies (7).

**Figure 1:**
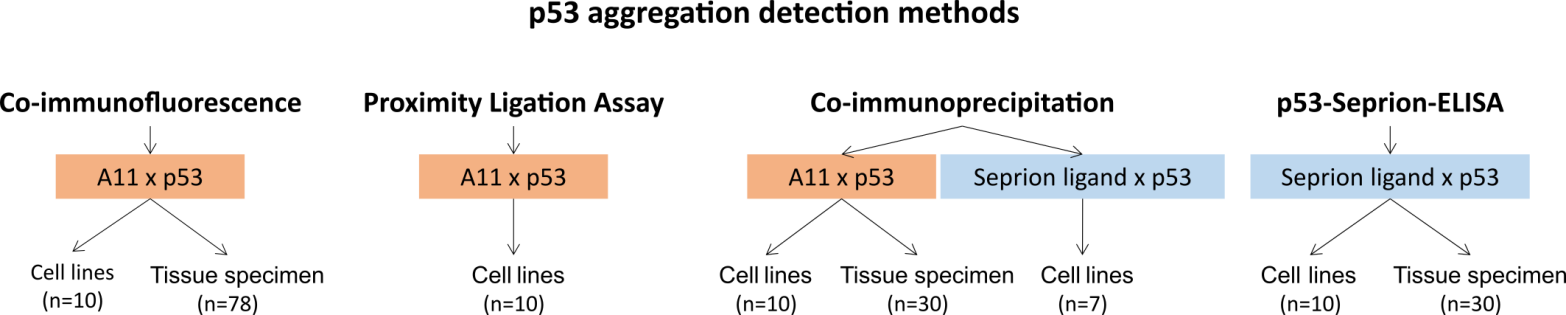
Overview of the p53 aggregation detection methods evaluated in this study.

In the present study, we aimed at comparing the state-of-the-art technique co-immunofluorescence (co-IF) with novel assays such as the proximity ligation assay (PLA), co-immunoprecipitation (co-IP), and the p53-Seprion-ELISA (**Figure 1**) in cancer cell lines and ovarian cancer tissues.

## Materials and methods

### Cell culture

Nine ovarian cancer cell lines (COV644, OAW42, COV318, COV362, ES2, OVCAR3, TYK-nu, 59M, and COV504) and one cervical cancer cell line (ME-180), either obtained from ATCC or kindly provided from Els Berns (Erasmus MC, Rotterdam, Netherlands) were grown in RPMI-1640 (Gibco/Life Technologies) supplemented with 10% FCS, 2 mM L-glutamine, 100 U/ml penicillin and 100 µg/ml streptomycin at 37°C in 5% CO_2_. STR-DNA-profile analysis was used for cell-line verification. In addition, *TP53* mutations specified for each cell line in the IARC TP53 Database were confirmed by Sanger sequencing (**Supplementary Table S1**).

### Patient cohort

Formalin-fixed paraffin-embedded (FFPE) tumor tissues from 78 patients diagnosed with ovarian cancer were collected at the Department of Obstetrics and Gynecology, Medical University of Innsbruck, Austria, and analyzed using co-IF. The study was approved by the local ethics committee (AN3507) and all patients gave their written informed consent. The *TP53* mutation status was identified by a functional yeast-based assay (FASAY) combined with Sanger sequencing as described previously (25, 26). Additionally, in a subgroup of 30 patients pulverized fresh-frozen tissue was available and therefore analyzed using co-IP and the p53-Seprion-ELISA.

### Immunofluorescence co-localization assay

The cancer cell lines were washed twice with PBS, fixed with 3.7% formaldehyde, and permeabilized with 0.3% Triton X-100 and 0.1% sodium citrate. Nonspecific antigenic sites were blocked using 5% BSA in PBS for 1h. Further, cells were labeled with primary antibodies: mouse anti-p53 antibody DO-1 (1:200, sc-126, Santa Cruz Biotechnology) and rabbit anti-amyloid oligomer A11 antibody (1:200, AB9234, Merck Millipore) or the anti-amyloid fibrils OC antibody (1:200, AB2286, Merck Millipore) for 2h at room temperature (RT) in a humidified chamber. Next, the cells were incubated with secondary anti-rabbit Alexa Fluor 488-conjugated (1:500, Life Technologies) and anti-mouse Alexa Fluor 568-conjugated (1:500, Life Technologies) antibodies for 1h at RT in the dark. Nuclear counterstain was done by incubation with DAPI (4’, 6-diamidino-2-phenylindole) solution. Between the blocking and staining steps, the cells were washed three times with PBS. The samples were analyzed using a laser scanning confocal microscope SP5 (Leica Microsystems).

FFPE tissues were deparaffinized in xylene and hydrated in decreasing concentrations of isopropanol. Briefly, antigen retrieval was performed by heat-induced epitope retrieval (HIER), while sections were immersed in 10 mM citrate buffer at pH 6.0 for two 5-minute intervals at 900 Watt using a microwave. To eliminate fixation-caused autofluorescence, sections were incubated in 1% sodium borohydride (Sigma Aldrich) three times. Nonspecific antigenic sites were blocked using 5% BSA/PBS. Next, sections were labeled with anti-p53 DO-1 (1:200) and anti-amyloid oligomer A11 (1:400) primary antibodies overnight in a humidified chamber at 4°C. The samples were incubated with secondary anti-rabbit Alexa Fluor 488-conjugated (1:750) and anti-mouse Alexa Fluor 568-conjugated (1:750) antibodies for 2h at RT in the dark. Nuclear counterstain was done by incubation with DAPI solution. Between the blocking and staining steps, the cells were washed three times with PBS.

All samples were analyzed using a laser scanning confocal microscope SP5 (Leica Microsystems).

### Proximity ligation assay

Cell lines were harvested, washed with PBS, and cytospins were prepared. The proximity ligation assay (PLA) was performed according to the manufacturer’s instructions using the Duolink PLA Kit (Sigma-Aldrich). To specifically detect p53 aggregates, the primary antibodies anti-p53 DO-1 (1:200) and the anti-amyloid oligomer A11 antibody (1:200) were used. Peripheral blood mononuclear cells (PBMCs) of a healthy individual were included as a negative control. The slides were analyzed using a LSM 780 confocal microscope (Zeiss).

### Co-immunoprecipitation and immunoblot

1,000,000 cells were seeded in Petri dishes and harvested at 80% of confluency. The cells as well as the tissue samples (approx. 15 mg) were lysed with 0.5% Triton X-100/PBS and incubated on ice for 1h. After sonication and centrifugation, the protein concentration of the supernatant was adjusted to 1 mg/ml. To prevent non-specific binding to the IP antibody, a lysate pre-purification step was performed with 1 mg/ml of the lysates. Therefore, the samples were incubated with 20 µl of Dynabeads^®^ Protein G (Life Technologies) for 1h at 4°C. For antibody binding, the pre-purified lysate was immunoprecipitated with the rabbit anti-amyloid oligomer A11 antibody (1:1000) overnight at 4°C. For the control reaction, the tissue lysates were incubated without the A11 antibody or only the lysis buffer with the A11 antibody. Then, the samples were incubated with 40 µl of Dynabeads^®^ Protein G 1h at 4°C. The beads were washed with lysis buffer, resuspended in 2x Laemmli buffer, and incubated at 95°C for 5 minutes. Samples were resolved by SDS-PAGE. Separated proteins were transferred to a nitrocellulose membrane. The membranes were blocked in Odyssey Blocking Buffer (LI-COR) and incubated with the mouse anti-p53 DO-1 primary antibody (1:200). Next, the membranes were labeled with anti-mouse IRDye^®^800 CW secondary antibody (LI-COR) and imaged using an Odyssey Scanner.

The Seprion-based co-immunoprecipitation was performed for 7 of 10 cancer cell lines (COV644, COV318, COV362, ES2, OVCAR3, TYK-nu, and COV504) and for 30 fresh-frozen OC samples. Crude lysates were incubated with Seprion-coated magnetic beads (Protein Aggregation Detection (PAD)-beads, Microsens Biotechnologies) according to the manufacturer’s instructions. Briefly, cell/tissue lysates were incubated with capture buffer and shaken by vibration at RT for 30 minutes. Beads were washed with wash buffers 1 and 2 and resuspended in 2x Laemmli buffer. Samples were resolved by SDS-PAGE as described above.

### p53-Seprion-ELISA

The aforementioned ten cancer cell lines were harvested at 70-80% confluency using Accutase (Sigma-Aldrich) and lysed with 1% Triton X-100/PBS for 30 minutes on ice. 12,500 cells per well were used as standard concentration. A 2.5% (w/v) lysate was prepared by lysing the pulverized tissues in the appropriate amount of ice-cold RIPA buffer complemented with protease inhibitor (Sigma-Aldrich). The lysates were incubated on ice for 5 minutes, immediately frozen on dry ice, and stored at -80°C until further analysis. All tissue samples were analyzed within 4 days after preparation.

The p53-Seprion-ELISA was performed as described previously (21). The tissue specimens were diluted 1:20 with ultrapure water and the anti-p53 (DO-1, Santa Cruz Biotechnologies) was used for the detection of p53 aggregates. All lysates were measured in triplicates and the average blank was subtracted from all sample replicates. The absorbance values were normalized to total protein concentration according to the recently published formula:


$$\frac{absorbance}{total\;protein}*1000$$

Samples with a p53 aggregation value below 1 were considered as negtive and samples with a value greater than 1 were considered as positive.

### Statistical analysis

The association between the categorical variables p53 protein expression, p53 aggregation, and histological subtypes of OC was determined using Cramer’s V and Fisher’s exact test. The level of significance was set at p< 0.05. All statistical analyses were performed using R Studio (version 4.0.3).

## Results

### Detection of p53 aggregates in cancer cell lines using co-immunofluorescence, proximity ligation assay, co-immunoprecipitation, and the p53-Seprion-ELISA

The aim of this study was to compare state-of-the-art co-immunofluorescence staining with novel technologies to detect p53 aggregates and assess the method’s applicability in cancer cell lines as well as tumor tissue specimens. Nine ovarian and one cervical cancer cell lines were evaluated by co-immunofluorescence (co-IF), proximity ligation assay (PLA), co-immunoprecipitation (co-IP), and the p53-Seprion-ELISA.

**Figure 2:**
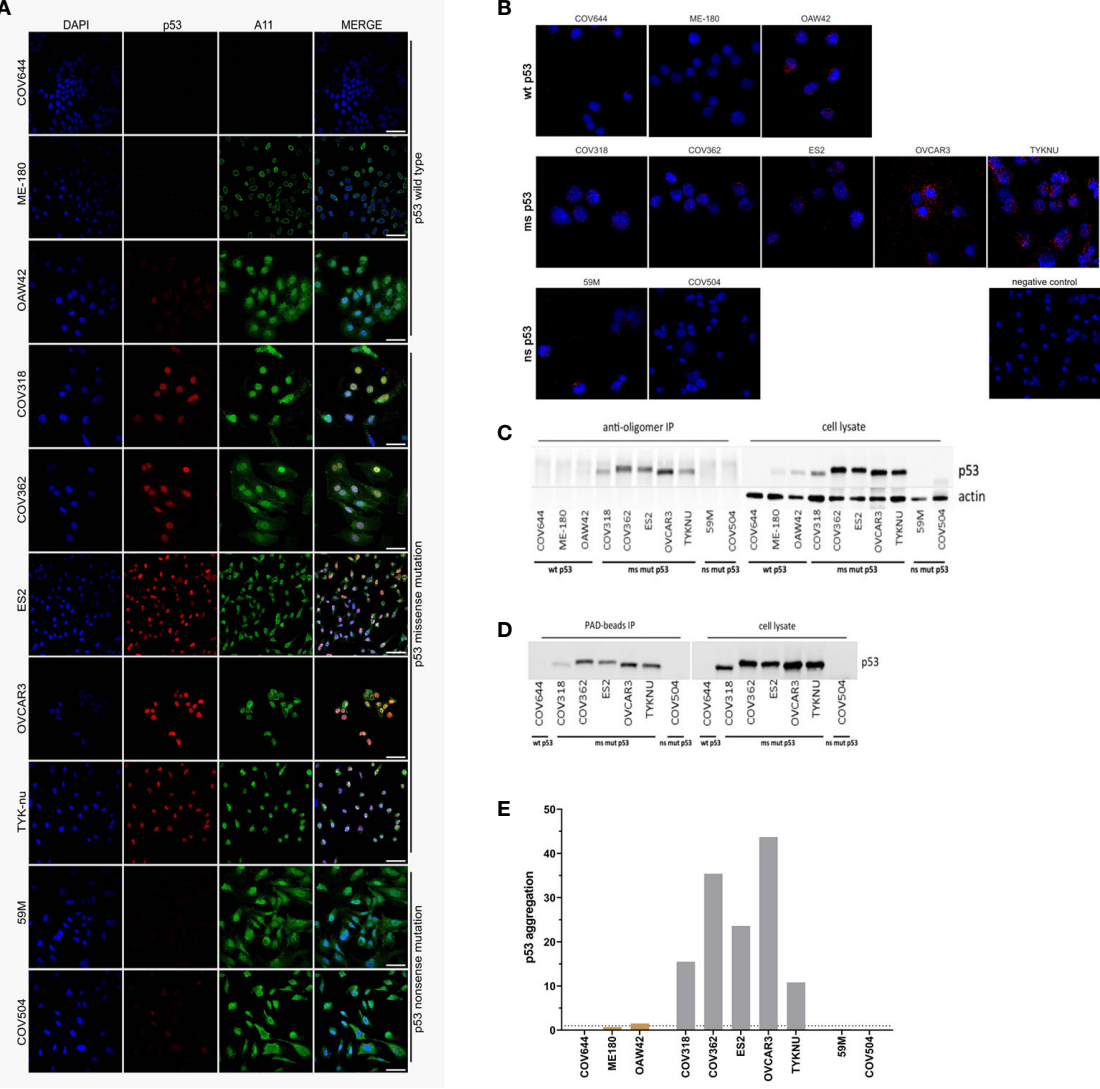
Detection of p53 aggregates in ovarian and cervical cancer cell lines. **(A)** Immunofluorescence co-localization assay (co-IF) using an anti-p53 (red) and an anti-oligomer (A11, green) antibody. Nuclear counterstaining was performed using DAPI. Scale bars: 50 µm. **(B)** Proximity ligation assay (PLA): red dots indicate p53 aggregates and nuclei in blue. **(C)** Co-immunoprecipitation (co-IP) was performed by using the anti-oligomer A11 antibody for the pull-down of amyloid proteins. Immunoblots for p53 were performed to show that p53 was present as oligomeric aggregates. **(D)** Amyloid proteins were immunoprecipitated with Seprion-coated beads (PAD-beads) and an immunoblot was performed to show that the aggregates consisted of p53. **(E)** The p53-Seprion-ELISA was performed to specifically detect p53 aggregates Dashed line, cut-off value for positive samples (p53 aggregation >1). Absorbance values were normalized to the total protein concentration. Grey, missense mutated cell lines; brown, wild-type p53 cell lines.

By using co-IF we were able to detect a strong co-localization of the p53 and the A11 antibody in the nucleus of all cell lines carrying a *TP53* missense mutation (**Figure 2A**). In the cell lines OAW42 (wild-type) and COV504 (frameshift (FS) deletion), only single cells showed co-localization of both antibodies. In the cell line COV644 (wild-type) neither p53 nor A11 expression was detected. In the remaining cell lines (ME-180, 59M) no p53 expression was detected, but they were found positive for the expression of amyloid proteins. In a subset of cell lines (ME-180, OAW42, COV318, COV362, ES2, OVCAR3, 59M, and COV504) the co-localization of p53 and amyloid fibrils, detected by the OC antibody, was evaluated. Again, in all missense mutated cell lines (COV318, COV362, ES2, and OVCAR3) co-localization of both antibodies was detected. In the wild-type (ME-180 and OAW42) and nonsense mutated (59M and COV504) cell lines only OC staining but no p53 staining was detected (**Supplementary Figure S1**).

In contrast to co-IF, the proximity ligation assay (PLA) allows determining whether the p53 and A11 antibodies bind in close proximity, demonstrating that p53 is present as oligomeric aggregates. In concordance with the co-IF results, we could observe PLA signals in all missense mutated cell lines (**Figure 2B**). The number of PLA dots varied considerably between the different cell lines, with the most signals observed in the OVCAR-3 and the TYK-nu cell lines. Intermediate levels were observed in the ES-2 and COV362 cell lines, and a weak signal in the COV318. In contrast to the co-IF results, the COV504 cell line was PLA negative, whereas the nonsense mutated 59M cell line, which was co-IF negative, resulted in a low amount of PLA signals in single cells.

The third method, co-IP, also allows the specific detection of aggregated p53. The A11 antibody was used to isolate the amyloid fractions, followed by immunoblotting to verify if p53 is present as oligomeric amyloid. Again, in all missense mutated cancer cell lines p53 aggregates could be detected to various extents (**Figure 2C**). The highest amount of p53 aggregates was detected in OVCAR3 and COV362 cell lines. In contrast to co-IF and PLA, no p53 aggregates were detected in wild-type or nonsense mutated cell lines. Furthermore, as an alternative to the A11 antibody, in seven cell lines, the Seprion ligand (PAD-beads) was applied to pull down the amyloid aggregates. The results of the Seprion-based co-IP were 100% concordant with the A11-based co-IP (**Figure 2D**).

Finally, the previously published p53-Seprion-ELISA was applied (**Figure 2E**). Consistently, p53 aggregates were detected in all missense mutated cell lines. The highest number of p53 aggregates was detected in the OVCAR3 and COV362 cell lines. None of the nonsense mutated cell lines and wild-type bearing cell lines, except the OAW42 cell line, formed p53 aggregates.

To sum up, all methods detected p53 aggregates in missense mutated cell lines (**Table 1**). The PLA was the only method detecting p53 aggregates in the 59M cell line, whereas only co-IF detected amyloid p53 in the COV504 cell line. In the OAW42 cell line all methods, except A11-based co-IP, showed the presence of p53 aggregates. In summary, in 7 out of 10 cell lines the applied methods showed 100% concordance.

**Table 1:** Comparison of the techniques applied in the detection of p53 aggregates *in vitro*.

Cell line	*TP53* status	Protein change	P53 aggregation detection method				
			co-IF	PLA	co-IP		p53-Seprion-ELISA
			(A11 x p53)	(A11 x p53)	(A11 x p53)	(Seprion ligand x p53)	(Seprion ligand x p53)
COV318	missense	I195F	+	+	+	+	15.5
COV362	missense	Y220C	+	+	++	++	35.4
ES2	missense	S241F	+	+	+	+	23.6
OVCAR-3	missense	R248Q	+	++	++	++	43.7
TYK-nu	missense	R175H	+	++	+	+	10.8
59M	FS deletion	H193KfsX49	–	+/-	–	n.e.	0.2
COV504	FS deletion	P322fsX13	+/-	–	–	–	0
COV644	WT	–	–	–	–	–	0.1
ME-180	WT	–	–	–	–	n.e.	0.7
OAW42	WT	–	+/-	+	–	n.e.	1.5
P53 aggregation positive			7/10	7/10	5/10	5/7	6/10

“-”, negative; “+/-”, only some of the cells show a (weak) signal; “+”, positive; “++”, strong signal; “n.e.”, not evaluated; “FS deletion”, frameshift deletion; “WT”, wild-type.

### Detection of p53 aggregates in ovarian cancer tumor tissue using three different methods

To validate our findings, co-IF, A11-based co-IP, and the p53-Seprion-ELISA were applied to detect p53 aggregates in ovarian cancer tissues. FFPE tissue specimens of 78 patients were analyzed using co-IF. Due to economic reasons (high costs, no tissue microarrays were available) the PLA was not applied on the FFPE samples. In a subset of 30 patients, fresh-frozen tissue was available; therefore, these patients were also analyzed using A11-based co-IP and the p53-Seprion-ELISA. The clinical pathological information of all patients is summarized in **Supplementary Table S2**. 51 out of 78 (65.4%) samples carried a *TP53* mutation. The most frequent mutations were missense mutations in 45 of 51 (88%) cases. In 5 of 51 (10%) cases FS deletions were present and in 1 of 51 (2%) cases a nonsense mutation was detected.

**Figure 3:**
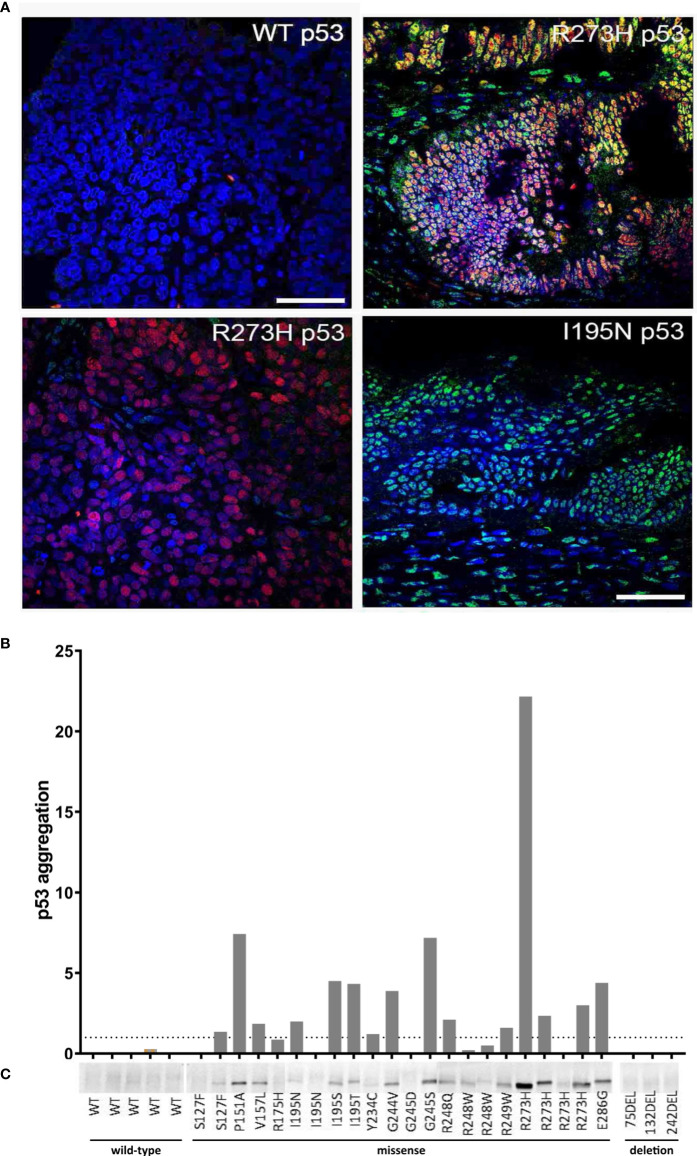
Detection of p53 aggregates in ovarian cancer (OC) tissue specimens. **(A)** Four representative OC FFPE tissue samples, which were analyzed by co-IF using an anti-p53 (red) and anti-amyloid (A11, green) antibody' are shown. Nuclear counterstaining was performed using DAPI. Scale bar = 50 µm. **(B)** Detection of p53 aggregates in 30 fresh-frozen OC tissue samples by using the p53-Seprion-ELISA. Absorbance values were normalized to the total protein concentration Dashed line, cut-off value for positive samples (p53 aggregation >1). **(C)** Co-IP was performed by using the anti-oligomer A11 antibody for the pull-down of amyloid proteins. Immunoblots for p53 were performed to show that the amyloid oligomers consisted of p53.

In 38 of 78 (48.7%) samples p53 aggregates could be detected by using co-IF (**Figure 3A**; **Supplementary Figure S2**, **Supplementary Table S3**). 33 out of 38 (87%) positive samples carried a *TP53* missense mutation, one sample a FS deletion, whereas the remaining four samples harbored wild-type p53. In 22 out of 38 (58%) positive samples strong co-localization in almost all cancer cells could be detected, whereas in the remaining 16 (42%) positive samples co-localization was detected in just a few cancer cells. Of note, there was a strong association with p53 protein expression (Cramer’s V = 0.787, Fisher’s p<0.001, **Supplementary Table S4**), however, 8 samples were p53 positive but A11 negative, suggesting that p53 protein expression did not necessarily lead to the formation of p53 aggregates. Moreover, we did not find a statistically significant association between histological subtypes and p53 protein expression or p53 aggregation (**Supplementary Table S5**).

The p53-Seprion-ELISA detected p53 aggregates in 15 of 30 (50%) fresh-frozen tissue samples (**Figure 3B**; **Supplementary Table S3** **)**. By using co-IP, p53 aggregates were detected in 17 of 30 (57%) fresh-frozen tissue samples, all of them harboring a *TP53* missense mutation (**Figure 3C**; **Supplementary Table S3**). Tumors with *TP53* wild-type or FS deletions were negative. Again, all of the positive samples carried a *TP53* missense mutation. Interestingly, in both assays, of four samples carrying the R273H mutation, one showed a very high aggregation level, two samples showed moderate p53 aggregation, and one sample was negative.

**Figure 4:**
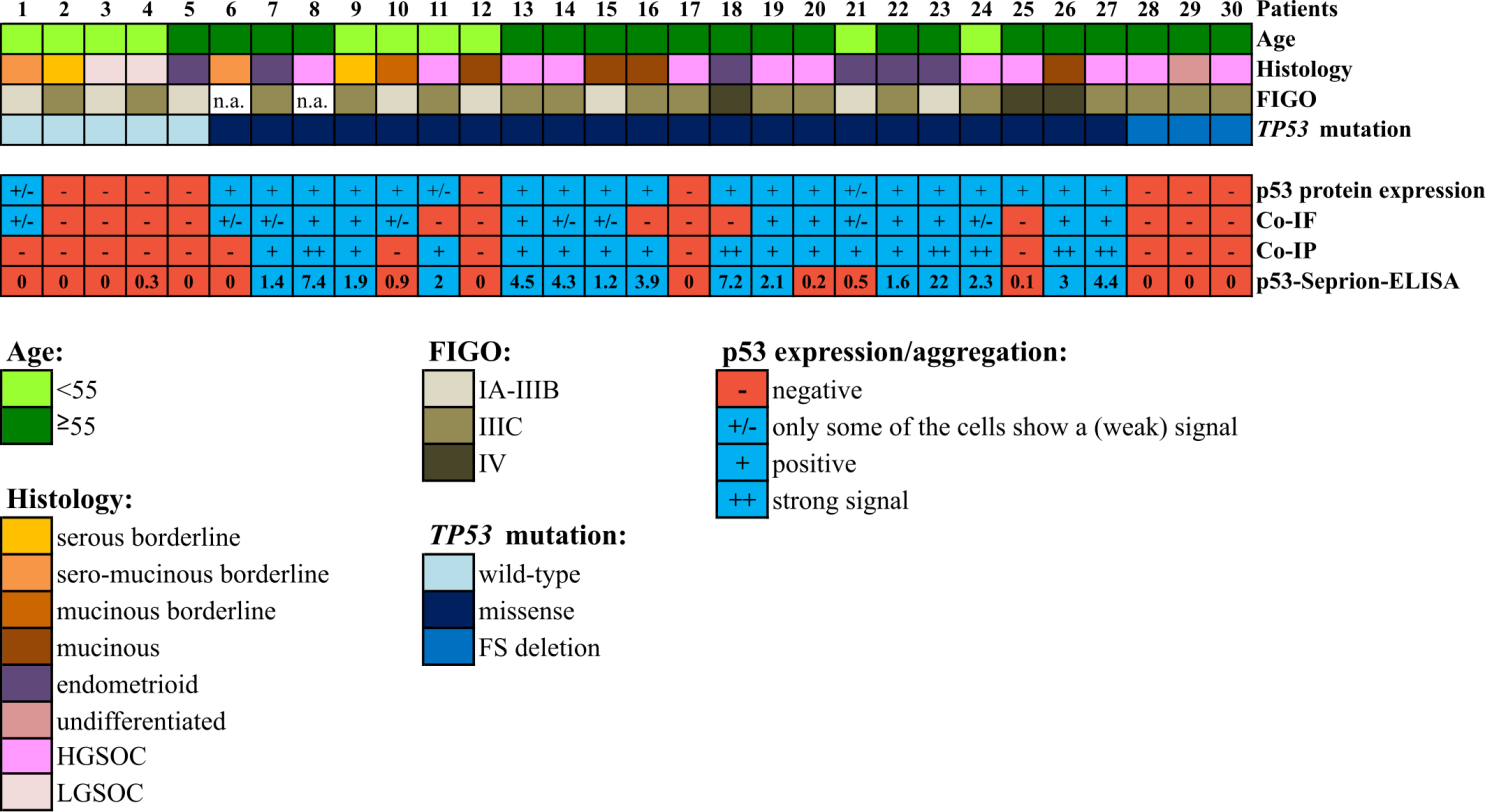
Comparison of the three techniques for the detection of p53 aggregates in 30 ovarian cancer tissue samples. Co-IF and co-IP were performed using the anti-p53 and the anti-oligomer A11 antibodies. “FIGO”, International Federation of Gynecology and Obstetrics; “Co-IF”, Immunofluorescence co-localization assay. “Co-IP”, Co-immunoprecipitation; “HGSOC”, high-grade serous ovarian cancer; “LGSOC”, low-grade serous ovarian cancer. “n.a.”; not available.

In summary, 100% concordance between all three methods could be achieved in 23 out of 30 (76.7%) samples. Co-IP and the p53-Seprion-ELISA, the methods that specifically detect p53 aggregates, showed concordance in 28 of 30 (93.3%) samples (**Figure 4**, **Supplementary Table S3**).

## Discussion

Highly sensitive and specific methods are the pre-requisites for the evaluation of p53 aggregates in patients’ biomaterials and interpretation of their clinical relevance. Our study provides a comprehensive comparison of four different technologies for the detection of p53 aggregates, involving state-of-the-art techniques as well as new innovative approaches (**Figure 1**). We were able to demonstrate 70% (cell lines) and 76.7% (tissue samples) concordance between optimized techniques.

Different conformational dyes and antibodies have been used to detect p53 aggregates by co-IF (5, 7, 27). Thioflavin and Congo Red are the traditional fluorescent dyes used for the detection of amyloid proteins. Nonetheless, both dyes have several limitations as they can cause false-positive results due to unspecific binding and Thioflavin does not detect amyloid oligomers and protofibrils (28). In our study, we focused on the A11 antibody that detects sequence-independent amyloid oligomers, but not monomers or fibrils, as with the OC antibody fewer p53 aggregates positive cell lines were identified (co-IF: OC: 4/8 positive cell lines vs A11: 7/10 positive cell lines; **Supplementary Figure S1** and **Figure 2A**). In addition, the novel Seprion ligand, previously used for the detection of PrP^Sc^, β-amyloid, α-synuclein, and huntingtin, was evaluated (22, 29–36). Co-IF, PLA, co-IP, and the p53-Seprion-ELISA showed 100% concordance in 7 of 10 (70%) cell lines (**Table 1**). Co-IF and PLA detected p53 aggregates in 7 of 10 cell lines, followed by the p53-Seprion ELISA (6/10), and A11-based co-IP (5/10). The Seprion-based co-IP resulted in 5/7 positive cell lines. When detecting p53 aggregates, it is critical to keep in mind that different types of amyloids are detected by the various conformation-dependent dyes, antibodies, and ligands. In the present study, the A11-based co-IF, PLA, and co-IP detect oligomer-like p53, while the Seprion-based approaches detect a wider range of amyloid proteins including also fibril-like p53 (**Table 2**). These differences might explain why we could not achieve 100% concordance between all applied techniques.

**Table 2:** Overview of methods for the detection of p53 aggregates in cell lines and tissue specimens.

	co-IF	PLA	co-IP	Seprion-based co-IP	p53-Seprion-ELISA
**Binding agents**	A11, p53 DO-1 antibody	A11, p53 DO-1 antibody	A11, p53 DO-1 antibody	Seprion ligand, p53 DO-1 antibody	Seprion ligand, p53 DO-1 antibody
**Principle**	Co-localization	Close proximity of two epitopes	Isolation of protein complexes	Isolation of protein complexes	Sandwich ELISA
**What is detected**	Amyloid p53 oligomers	Amyloid p53 oligomers	Amyloid p53 oligomers	Amyloid p53 oligomers, proto-fibrils, and fibrils	Amyloid p53 oligomers, proto-fibrils, and fibrils
**Sample type**	FFPE, cytopreparations	FFPE, cytopreparations	Fresh-frozen tissue and cell line lysates	Fresh-frozen tissue and cell line lysates	Fresh-frozen tissue and cell line lysates
**Ease of use**	Experience in fluorescence microscopy needed	Experience in fluorescence microscopy needed, Quantification of PLA dots requires either a scanning microscope with appropriate software or high-resolution images and subsequent analysis software (ImageJ, CellProfiler, …)	Easy	Easy	Very easy
**Large scale**	Only if applied on tissue microarrays	Only if applied on tissue microarrays	no	no	yes
**Advantage**	Use on FFPE, low costs, allows single-cell analysis	Use on FFPE, high sensitivity, high specificity, quantification, allows single-cell analysis	Semi-quantitative, high specificity	Semi-quantitative, high specificity	Quantification, high-throughput, high sensitivity, high reproducibility
**Disadvantage**	No quantification, limited specificity	Time-consuming, high costs	Fresh-frozen tissue needed, time-consuming, complex procedure, no single-cell analysis	Fresh-frozen tissue needed, time-consuming, complex procedure, no single-cell analysis	Fresh-frozen tissue needed, no single-cell analysis

To evaluate the applicability of these methods in patient samples, primary ovarian cancer tissues were analyzed using co-IF, A11-based co-IP, and the p53-Seprion-ELISA. Although the PLA resulted in a high detection rate in the cell lines and would be the method of choice for the analysis of FFPE tissues, its application is rather expensive on large tissue sections and tissue microarrays should be the preferred sample type; however, these were not available. We show concordant results between the three techniques in 76.7% of samples and the p53 aggregates detection rate ranged from 48.7% to 56.7%. All positive samples harbored a *TP53* missense mutation. Additionally, co-IF detected four positive wild-type samples and one positive sample with a FS deletion. Moreover, in our study, the R273H missense mutation showed differences in the ability to form amyloid-like structures in four ovarian cancer patients. For this mutation, a high propensity to form aggregates was reported previously in breast cancer (5). The positive wild-type samples as well as the varying p53 aggregation levels in patients with identical *TP53* mutation suggest that a missense mutation alone is not sufficient to increase the capability of p53 to form aggregates. It has been shown that inhibition of MDM2-mediated p53 degradation promoted the formation of wild-type p53 aggregates (12). Molecular interaction partners of p53 may also enhance the aggregation propensity of p53. For example, the transient interaction between mutant p53 (R175H) and the cellular chaperone heat shock protein 70 (HSP70) resulted in the increased half-life of mutant p53 and exposure of an aggregation-prone region. In the presence of MDM2, these two proteins can form amyloid-like aggregates (37). Furthermore, the heat shock protein 90 (HSP90) has been reported to interact with the p53 DNA-binding domain, leading to a structural change in the protein and the formation of a molten globule state, which is prone to aggregation (38). Additionally, the expression of specific p53 isoforms may have an impact on the capability of p53 to form amyloid-like structures as well. The wild-type Δ133p53β isoform has been shown to form aggregates in cancer cells and tumor biopsies (39). Moreover, the Δ40p53 isoform, which lacks the p53 transactivation domain, has been reported to have a high aggregation tendency in endometrial cancer cells (40).

The cell lines ME-180 and 59M as well as some of the tissue samples pointed out the limitations of the state-of-the-art co-IF and the need for more specific novel methods. These samples were p53 negative, but still showed abundant A11 staining (**Figure 2A**, **Figure 3A**), demonstrating that other amyloid proteins are present and co-localization of p53 and A11 antibodies in the same subcellular compartment does not necessarily mean that p53 aggregates are detected. The novel proximity ligation assay (PLA) is a powerful tool for the highly specific p53 aggregate detection as it only results in a fluorescent signal when the p53 and A11 antibodies are bound in close proximity indicating the presence of p53 aggregates. Another major advantage of the method is that the individual PLA dots can be quantified using freely available software tools such as ImageJ or CellProfiler (41–43). Moreover, the PLA can be performed on formalin-fixed paraffin-embedded (FFPE) tissue samples, which are often the only available source of clinical samples, whereas co-IP and ELISA are limited to fresh-frozen material. The disadvantages of the PLA are the extensive costs if tissue microarrays are not available and the relatively long duration of the procedure of 2 days. In contrast, the p53-Seprion-ELISA has a turnaround time of only 5 hours and allows high-throughput analysis, but requires fresh-frozen tissue, which is often not archived in clinical routine.

In conclusion, we compared the state-of-the-art p53 aggregation detection method co-IF with co-IP and the novel PLA and Seprion technology. The PLA and p53-Seprion-ELISA are the only two methods allowing quantitative measurement of p53 aggregates. Taking into consideration that the most widely available source of tumor tissue is formalin-fixed and paraffin-embedded, the PLA outperforms co-IF in terms of sensitivity, specificity, and quantification of p53 aggregates. Wherever fresh-frozen material is available, the p53-Seprion-ELISA should be preferred as it allows rapid, high-throughput testing in contrast to co-IP. Our study provides the basis for the reliable detection of p53 aggregates in biological specimens to unravel the clinical significance of p53 aggregates, especially since potential p53-aggregation targeting drugs are currently under investigation and would open up new paths in cancer therapy. Moreover, mutated p53 is not the only tumor suppressor protein with enhanced aggregation tendency. In silico analyses demonstrated that protein aggregation is not a rare phenomenon, but far more common, and other tumor suppressor proteins, such as PTEN or Axin, have been identified to form amyloid-like structures (44–47). Our herein mentioned tools can be easily adapted to detect other types of amyloid-like proteins and help to evaluate their biological and clinical relevance in various cancer types.

## Data availability statement

The raw data supporting the conclusions of this article will be made available by the authors, without undue reservation.

## Ethics statement

The studies involving human participants were reviewed and approved by Ethics Committee of the Medical University of Innsbruck. The patients/participants provided their written informed consent to participate in this study.

## Author contributions

RZ and NC designed the study. NH, KK, EM, AB, and EP performed the experiments and collected and interpreted the data. HF provided the clinical pathological information for all patients. NH, KK, and EM prepared the figures and tables, and wrote the manuscript. NH, KK, EM, AB, EP, HF, AZ, CM, RZ, and NC reviewed and revised the manuscript. All authors read and approved the final version of the manuscript.

## Funding

Our work was supported by the Seventh Framework Programme (FP7) project of the European Union called “Ganetespib in metastatic, p53-mutant ovarian cancer – GANNET53” (grant agreement no. 602602).

## Acknowledgments

The authors thank Dr. Martin Offterdinger from the Core Facility Biooptics, Innsbruck Medical University for his assistance and expertise, and Els Berns (Erasmus MC, Rotterdam, Netherlands) for kindly providing some of the cell lines used in this study. This study received support from Microsens Biotechnologies (London, UK) in the form of an in-kind contribution of Seprion-coated microplates and capture buffer.

## Conflict of interest

The authors declare that the research was conducted in the absence of any commercial or financial relationships that could be construed as a potential conflict of interest.

The handling editor EB declared a shared research group with the authors NC and RZ at the time of review.

## Publisher’s note

All claims expressed in this article are solely those of the authors and do not necessarily represent those of their affiliated organizations, or those of the publisher, the editors and the reviewers. Any product that may be evaluated in this article, or claim that may be made by its manufacturer, is not guaranteed or endorsed by the publisher.

## References

[1] Chiti F Dobson CM . Protein misfolding, functional amyloid, and human disease. Annu Rev Biochem (2006) 75:333–66. doi: 10.1146/annurev.biochem.75.101304.123901 16756495

[2] Scheckel C Aguzzi A . Prions, prionoids and protein misfolding disorders. Nat Rev Genet (2018) 19(7):405–18. doi: 10.1038/s41576-018-0011-4 29713012

[3] Xu J Reumers J Couceiro JR De Smet F Gallardo R Rudyak S . Gain of function of mutant p53 by coaggregation with multiple tumor suppressors. Nat Chem Biol (2011) 7(5):285–95. doi: 10.1038/nchembio.546 21445056

[4] Soragni A Janzen DM Johnson LM Lindgren AG Thai-Quynh Nguyen A Tiourin E . A designed inhibitor of p53 aggregation rescues p53 tumor suppression in ovarian carcinomas. Cancer Cell (2016) 29(1):90–103. doi: 10.1016/j.ccell.2015.12.002 26748848 PMC4733364

[5] Levy CB Stumbo AC Ano Bom AP Portari EA Cordeiro Y Silva JL . Co-Localization of mutant p53 and amyloid-like protein aggregates in breast tumors. Int J Biochem Cell Biol (2011) 43(1):60–4. doi: 10.1016/j.biocel.2010.10.017 21056685

[6] Yang-Hartwich Y Bingham J Garofalo F Alvero AB Mor G . Detection of p53 protein aggregation in cancer cell lines and tumor samples. Methods Mol Biol (2015) 1219:75–86. doi: 10.1007/978-1-4939-1661-0_7 25308263

[7] De Smet F Saiz Rubio M Hompes D Naus E De Baets G Langenberg T . Nuclear inclusion bodies of mutant and wild-type p53 in cancer: a hallmark of p53 inactivation and proteostasis remodelling by p53 aggregation. J pathology. (2017) 242(1):24–38. doi: 10.1002/path.4872 28035683

[8] Ghosh S Salot S Sengupta S Navalkar A Ghosh D Jacob R . p53 amyloid formation leading to its loss of function: implications in cancer pathogenesis. Cell Death differentiation. (2017) 24(10):1784–98. doi: 10.1038/cdd.2017.105 PMC5596421 28644435

[9] Silva JL Rangel LP Costa DC Cordeiro Y De Moura Gallo CV . Expanding the prion concept to cancer biology: dominant-negative effect of aggregates of mutant p53 tumour suppressor. Biosci Rep. (2013) 33(4):e00054. doi: 10.1042/BSR20130065 24003888 PMC3728989

[10] Ano Bom AP Rangel LP Costa DC de Oliveira GA Sanches D Braga CA . Mutant p53 aggregates into prion-like amyloid oligomers and fibrils: implications for cancer. J Biol Chem (2012) 287(33):28152–62. doi: 10.1074/jbc.M112.340638 PMC3431633 22715097

[11] Zhang Y Cao L Nguyen D Lu H . TP53 mutations in epithelial ovarian cancer. Trans Cancer Res (2016) 5(6):650–63. doi: 10.21037/tcr.2016.08.40 PMC6320227 30613473

[12] Yang-Hartwich Y Soteras MG Lin ZP Holmberg J Sumi N Craveiro V . p53 protein aggregation promotes platinum resistance in ovarian cancer. Oncogene. (2015) 34(27):3605–16. doi: 10.1038/onc.2014.296 25263447

[13] Zhang Y Hu Y Wang JL Yao H Wang H Liang L . Proteomic identification of ERP29 as a key chemoresistant factor activated by the aggregating p53 mutant Arg282Trp. Oncogene. (2017) 36(39):5473–83. doi: 10.1038/onc.2017.152 28534505

[14] Neal A Lai T Singh T Rahseparian N Grogan T Elashoff D . Combining ReACp53 with carboplatin to target high-grade serous ovarian cancers. Cancers. (2021) 13(23):5908. doi: 10.3390/cancers13235908 34885017 PMC8657291

[15] Ferraz da Costa DC Campos NPC Santos RA Guedes-da-Silva FH Martins-Dinis MMDC Zanphorlin L . Resveratrol prevents p53 aggregation *in vitro* and in breast cancer cells. Oncotarget. (2018) 9(49):29112–22. doi: 10.18632/oncotarget.25631 PMC6044377 30018739

[16] Rangel LP Ferretti GDS Costa CL Andrade S Carvalho RS Costa DCF . p53 reactivation with induction of massive apoptosis-1 (PRIMA-1) inhibits amyloid aggregation of mutant p53 in cancer cells. J Biol Chem (2019) 294(10):3670–82. doi: 10.1074/jbc.RA118.004671 PMC6416452 30602570

[17] Miller JJ Blanchet A Orvain C Nouchikian L Reviriot Y Clarke RM . Bifunctional ligand design for modulating mutant p53 aggregation in cancer. Chem science. (2019) 10(46):10802–14. doi: 10.1039/C9SC04151F PMC7006507 32055386

[18] Farmer KM Ghag G Puangmalai N Montalbano M Bhatt N Kayed R . P53 aggregation, interactions with tau, and impaired DNA damage response in alzheimer's disease. Acta neuropathologica Commun (2020) 8(1):132. doi: 10.1186/s40478-020-01012-6 PMC7418370 32778161

[19] Kayed R Head E Sarsoza F Saing T Cotman CW Necula M . Fibril specific, conformation dependent antibodies recognize a generic epitope common to amyloid fibrils and fibrillar oligomers that is absent in prefibrillar oligomers. Mol Neurodegener. (2007) 2:18. doi: 10.1186/1750-1326-2-18 17897471 PMC2100048

[20] Iwahashi N Ikezaki M Nishikawa T Namba N Ohgita T Saito H . Sulfated glycosaminoglycans mediate prion-like behavior of p53 aggregates. Proc Natl Acad Sci United States America. (2020) 117(52):33225–34. doi: 10.1073/pnas.2009931117 PMC7776818 33318190

[21] Maritschnegg E Heinzl N Wilson S Deycmar S Niebuhr M Klameth L . Polymer-Ligand-Based ELISA for robust, high-throughput, quantitative detection of p53 aggregates. Analytical Chem (2018) 90(22):13273–9. doi: 10.1021/acs.analchem.8b02373 30277755

[22] Sathasivam K Lane A Legleiter J Warley A Woodman B Finkbeiner S . Identical oligomeric and fibrillar structures captured from the brains of R6/2 and knock-in mouse models of huntington's disease. Hum Mol Genet (2010) 19(1):65–78. doi: 10.1093/hmg/ddp467 19825844 PMC2792149

[23] Lane A Stanley CJ Dealler S Wilson SM . Polymeric ligands with specificity for aggregated prion proteins. Clin Chem (2003) 49(10):1774–5. doi: 10.1373/49.10.1774

[24] Söderberg O Gullberg M Jarvius M Ridderstråle K Leuchowius K-J Jarvius J . Direct observation of individual endogenous protein complexes *in situ* by proximity ligation. Nat Methods (2006) 3(12):995–1000. doi: 10.1038/nmeth947 17072308

[25] Concin N Becker K Slade N Erster S Mueller-Holzner E Ulmer H . Transdominant DeltaTAp73 isoforms are frequently up-regulated in ovarian cancer. evidence for their role as epigenetic p53 inhibitors *in vivo* . Cancer Res (2004) 64(7):2449–60. doi: 10.1158/0008-5472.can-03-1060 15059898

[26] Concin N Hofstetter G Berger A Gehmacher A Reimer D Watrowski R . Clinical relevance of dominant-negative p73 isoforms for responsiveness to chemotherapy and survival in ovarian cancer: evidence for a crucial p53-p73 cross-talk *in vivo* . Clin Cancer Res an Off J Am Assoc Cancer Res (2005) 11(23):8372–83. doi: 10.1158/1078-0432.CCR-05-0899 16322298

[27] Lasagna-Reeves CA Clos AL Castillo-Carranza D Sengupta U Guerrero-Muñoz M Kelly B . Dual role of p53 amyloid formation in cancer; loss of function and gain of toxicity. Biochem Biophys Res Commun (2013) 430(3):963–8. doi: 10.1016/j.bbrc.2012.11.130 23261448

[28] Yakupova EI Bobyleva LG Vikhlyantsev IM Bobylev AG . Congo Red and amyloids: history and relationship. Bioscience Rep (2019) 39(1):BSR20181415. doi: 10.1042/BSR20181415 PMC6331669 30567726

[29] Bobrowska A Donmez G Weiss A Guarente L Bates G . SIRT2 ablation has no effect on tubulin acetylation in brain, cholesterol biosynthesis or the progression of huntington's disease phenotypes *in vivo* . PLoS One (2012) 7(4):e34805. doi: 10.1371/journal.pone.0034805 22511966 PMC3325254

[30] Edwards JC Moore SJ Hawthorn JA Neale MH Terry LA . PrP(Sc) is associated with b cells in the blood of scrapie-infected sheep. Virology. (2010) 405(1):110–9. doi: 10.1016/j.virol.2010.05.023 20646730

[31] González L Horton R Ramsay D Toomik R Leathers V Tonelli Q . Adaptation and evaluation of a rapid test for the diagnosis of sheep scrapie in samples of rectal mucosa. J Veterinary Diagn Invest (2008) 20(2):203–8. doi: 10.1177/104063870802000209 18319433

[32] Labbadia J Novoselov SS Bett JS Weiss A Paganetti P Bates GP . Suppression of protein aggregation by chaperone modification of high molecular weight complexes. Brain J Neurol (2012) 135(Pt 4):1180–96. doi: 10.1093/brain/aws022 PMC3326252 22396390

[33] Mielcarek M Benn CL Franklin SA Smith DL Woodman B Marks PA . SAHA decreases HDAC 2 and 4 levels *in vivo* and improves molecular phenotypes in the R6/2 mouse model of huntington's disease. PLoS One (2011) 6(11):e27746. doi: 10.1371/journal.pone.0027746 22140466 PMC3225376

[34] Moumne L Campbell K Howland D Ouyang Y Bates GP . Genetic knock-down of HDAC3 does not modify disease-related phenotypes in a mouse model of huntington's disease. PLoS One (2012) 7(2):e31080. doi: 10.1371/journal.pone.0031080 22347433 PMC3275566

[35] Terry LA Howells L Hawthorn J Edwards JC Moore SJ Bellworthy SJ . Detection of PrPsc in blood from sheep infected with the scrapie and bovine spongiform encephalopathy agents. J virology. (2009) 83(23):12552–8. doi: 10.1128/JVI.00311-09 PMC2786716 19740979

[36] Lane AR Stanley CJ Wilson SM . Binding of aggregated forms of proteins. Google Patents (2014):US Patent No. US20120009595A1.

[37] Wiech M Olszewski MB Tracz-Gaszewska Z Wawrzynow B Zylicz M Zylicz A . Molecular mechanism of mutant p53 stabilization: The role of HSP70 and MDM2. PLoS One (2012) 7(12):e51426. doi: 10.1371/journal.pone.0051426 23251530 PMC3520893

[38] Park SJ Borin BN Martinez-Yamout MA Dyson HJ . The client protein p53 forms a molten globule-like state in the presence of Hsp90. Nat Struct Mol Biol (2011) 18(5):537–41. doi: 10.1038/nsmb.2045 PMC3087862 21460846

[39] Arsic N Slatter T Gadea G Villain E Fournet A Kazantseva M . Δ133p53β isoform pro-invasive activity is regulated through an aggregation-dependent mechanism in cancer cells. Nat Commun (2021) 12(1):5463. doi: 10.1038/s41467-021-25550-2 34526502 PMC8443592

[40] Melo Dos Santos N de Oliveira GAP Ramos Rocha M Pedrote MM Diniz da Silva Ferretti G Pereira Rangel L . Loss of the p53 transactivation domain results in high amyloid aggregation of the Δ40p53 isoform in endometrial carcinoma cells. J Biol Chem (2019) 294(24):9430–9. doi: 10.1074/jbc.RA119.007566 PMC6579457 31028175

[41] Klaesson A Grannas K Ebai T Heldin J Koos B Leino M . Improved efficiency of *in situ* protein analysis by proximity ligation using UnFold probes. Sci Rep (2018) 8(1):5400. doi: 10.1038/s41598-018-23582-1 29599435 PMC5876389

[42] Carpenter AE Jones TR Lamprecht MR Clarke C Kang IH Friman O . CellProfiler: image analysis software for identifying and quantifying cell phenotypes. Genome Biol (2006) 7(10):R100. doi: 10.1186/gb-2006-7-10-r100 17076895 PMC1794559

[43] Schneider CA Rasband WS Eliceiri KW . NIH Image to ImageJ: 25 years of image analysis. Nat Methods (2012) 9(7):671–5. doi: 10.1038/nmeth.2089 PMC5554542 22930834

[44] Anvarian Z Nojima H van Kappel EC Madl T Spit M Viertler M . Axin cancer mutants form nanoaggregates to rewire the wnt signaling network. Nat Struct Mol Biol (2016) 23(4):324–32. doi: 10.1038/nsmb.3191 26974125

[45] Claes F Maritschnegg E De Baets G Siekierska A Rubio MS Ramakers M . The tumor suppressor protein PTEN undergoes amyloid-like aggregation in tumor cells. bioRxiv (2020) Available at: https://https://www.biorxiv.org/content/10.1101/2020.11.30.402115v1 doi: 10.1101/2020.11.30.402115

[46] De Baets G Van Doorn L Rousseau F Schymkowitz J . Increased aggregation is more frequently associated to human disease-associated mutations than to neutral polymorphisms. PLoS Comput Biol (2015) 11(9):e1004374. doi: 10.1371/journal.pcbi.1004374 26340370 PMC4560525

[47] Housmans JAJ Wu G Schymkowitz J Rousseau F . A guide to studying protein aggregation. FEBS J (2021). doi: 10.1111/febs.16312 34862849

