# Supplementary material for: A comparison of four technologies for detecting p53 aggregates in ovarian cancer
Source: Front Oncol. 2022 Sep 8;12:976725. doi: 10.3389/fonc.2022.976725 (PMC9493009; doi:10.3389/fonc.2022.976725)
Supplement: Supplementary file 1 [file DataSheet_1.pdf]

Supplementary Material

1 Supplementary Figures

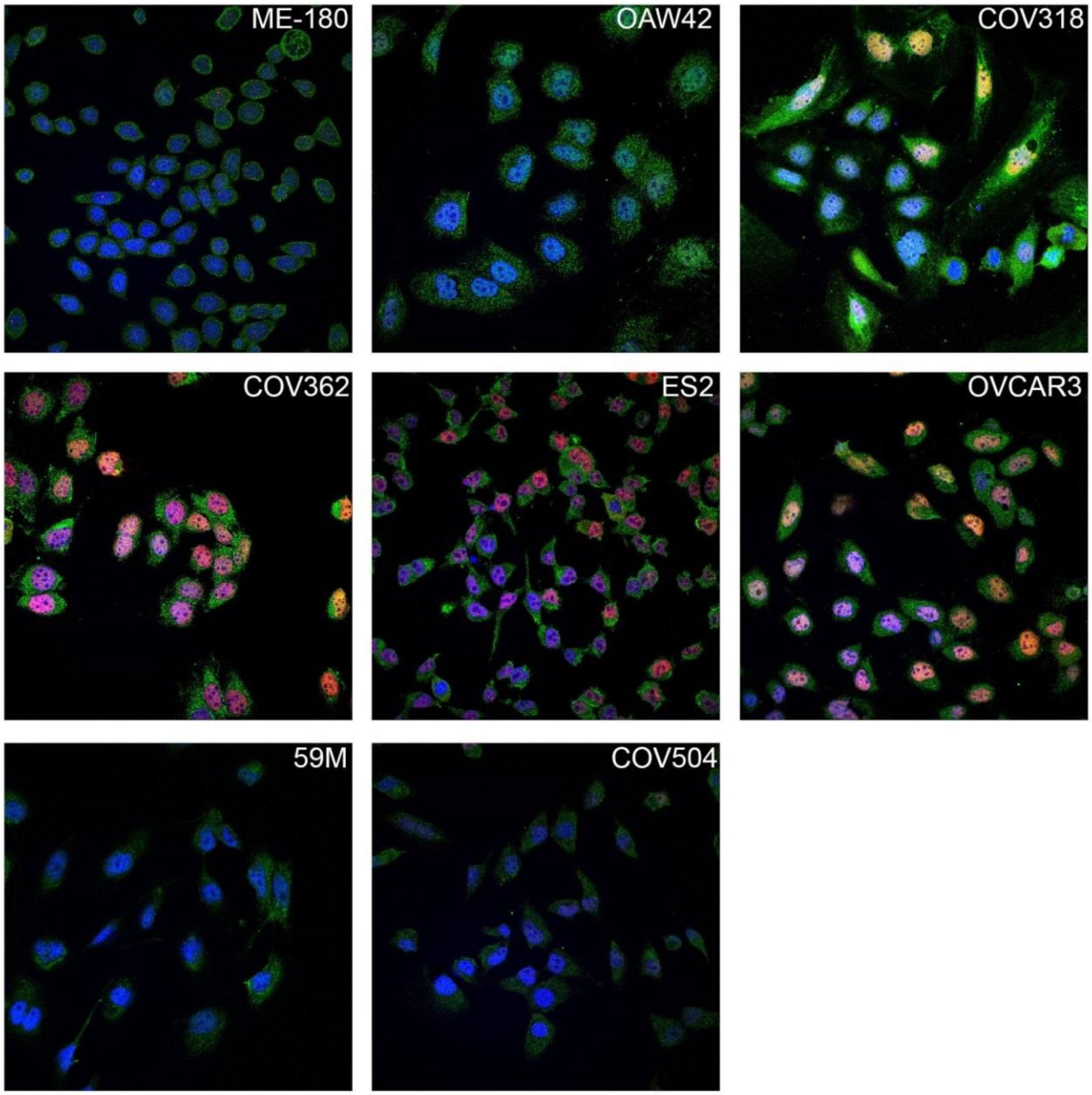

Supplementary Figure 1. Detection of p53 and amyloid fibrils (OC antibody) in ovarian cancer cell lines by co-IF. Nuclear counterstain has been achieved by incubation with DAPI solution. The images were acquired by SP5 Leica microscope.

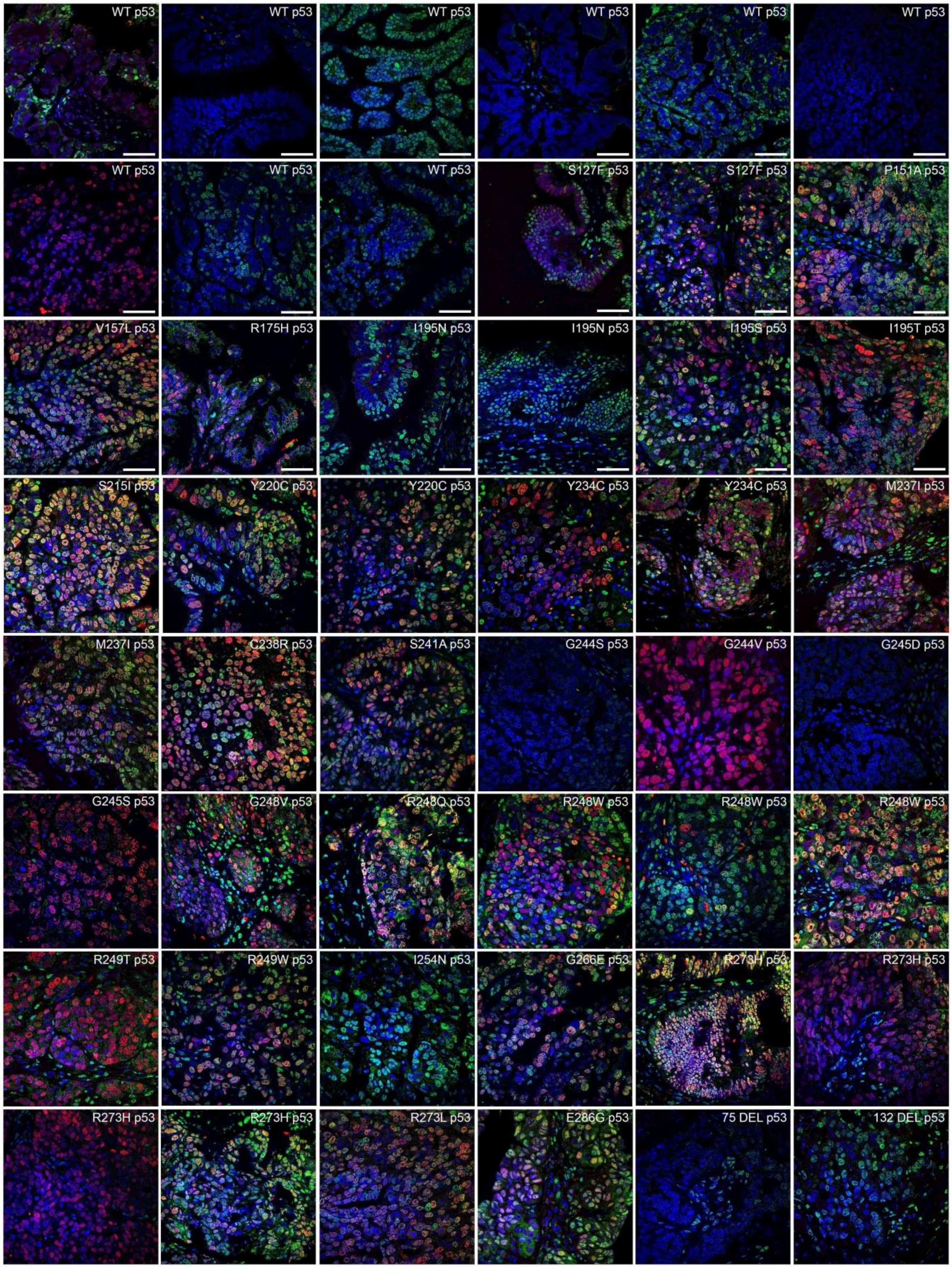

Supplementary Figure 2: Detection of p53 and amyloid oligomer in paraffin-embedded ovarian cancer tissue by immunofluorescence co-localization assay. The samples were labeled with anti-p53 DO1 and anti-oligomer A11 antibodies. Nuclear counterstain has been achieved by incubation with DAPI solution. The images were acquired by SP5 Leica microscope; scale bars: 50 µm.

2 Supplementary Tables

Supplementary Table 1: Characteristics of the 10 cell lines used in this study.

Cell line	Origin[a]	*TP53* status[b]	Protein change[b]	cDNA description[b]
**COV644**	Ovarian mucinous adenocarcinoma	WT	-	-
**ME-180**	Human-papillomavirus-related cervical squamous-cell carcinoma derived from metastatic site: omentum	WT	-	-
**OAW42**	Ovarian serous cystadenocarcinoma derived from metastatic site: ascites	WT	-	-
**COV318**	High-grade ovarian serous adenocarcinoma derived from metastatic site: ascites	missense	I195F	c.583A>T
**COV362**	High-grade ovarian adenocarcinoma derived from metastatic site: pleural effusion	missense	Y220C	c.659A>G
**ES2**	Ovarian clear-cell adenocarcinoma	missense	S241F	c.722C>T
**OVCAR3**	High-grade ovarian serous adenocarcinoma derived from metastatic site: ascites	missense	R248Q	c.743G>A
**TYK-nu**	High-grade ovarian serous adenocarcinoma	missense	R175H	c.524G>A
**59M**	High-grade ovarian serous adenocarcinoma derived from metastatic site: ascites	FS deletion	H193KfsX49	c.577_592del
**COV504**	Ovarian carcinoma derived from metastatic site: pleural effusion	FS deletion	P322fsX13	c.965_966del

[a] Source: Cellosaurus (https://web.expasy.org/cellosaurus/, accessed on 11/07/2022, (1)).
[b] Source: The *TP53* Database (R20, July 2019, https://tp53.isb-cgc.org/, accessed on 11/07/2022, (2))

Supplementary Table 2: Baseline clinical-pathological characteristics of the study cohort.

Characteristics	n (%)	n (%)
**Total**	78 (100.0%)	30 (100%)
**Age (years)**		
**Median**	59	63
**Range**	24 - 82	24 - 82
**< 55 years**	32 (41.0%)	10 (33.3%)
**≥ 55 years**	46 (59.0%)	20 (66.7%)
**Histology**		
**HGSOC**	28 (35.9%)	12 (40.0%)
**LGSOC**	6 (7.7%)	2 (6.7%)
**Endometrioid**	16 (20.5%)	6 (20.0%)
**Mucinous**	8 (10.3%)	4 (13.3%)
**Mucinous borderline**	2 (2.36%)	1 (3.3%)
**Sero-mucinous borderline**	3 (3.8%)	2 (6.7%)
**Serous borderline**	6 (7.7%)	2 (6.7%)
**Undifferentiated**	8 (10.3%)	1 (3.3%)
**Carcinosarcoma**	1 (1.3%)	0 (0%)
**FIGO**		
**IA-IIIB**	26 (33.3%)	8 (26.7%)
**IIIC**	39 (50.0%)	17 (56.7%)
IV	10 (12.8%)	3 (10.0%)
**Missing**	3 (3.8%)	2 (6.7%)
***TP53* mutation status**		
**Missense**	45 (57.7%)	22 (73.3%)
**FS deletion**	5 (6.4%)	3 (10.0%)
**Nonsense**	1 (1.3%)	0 (0%)
**Wild-type**	27 (34.6%)	5 (16.7%)

Supplementary Table 3: Comparison of the three techniques for the detection of p53 aggregates in the ovarian cancer tissue samples.

Patient ID	*TP53* status	Protein change	Histological subtype	p53 protein expression (IF)	P53 aggregation detection method		
					co-IF	co-IP	p53-Seprion-ELISA
					(A11 x p53)	(A11 x p53)	(Seprion ligand x p53)
**553**	WT	-	sero-mucinous borderline	+/-	+/-	-	0
**1698**	WT	-	serous borderline	-	-	-	0
**1281**	WT	-	LGSOC	-	-	-	0
**3306**	WT	-	LGSOC	-	-	-	0.28
**3179**	WT	-	endometrioid	-	-	-	0
**2131**	missense	S127F	sero-mucinous borderline	+	+/-	-	0
**2132**	missense	S127F	endometrioid	+	+/-	+	1.35
**2188**	missense	P151A	HGSOC	+	+	++	7.42
**2648**	missense	V157L	serous borderline	+	+	+	1.85
**1234**	missense	R175H	mucinous borderline	+	+/-	-	0.86
**1181**	missense	I195N	HGSOC	+/-	-	+	1.99
**2228**	missense	I195N	mucinous	-	-	-	0
**1930**	missense	I195S	HGSOC	+	+	+	4.5
**2037**	missense	I195T	HGSOC	+	+/-	+	4.32
**1821**	missense	Y234C	mucinous	+/-	+/-	+	1.21
**1644**	missense	G244V	mucinous	+	-	+	3.88
**2683**	missense	G245D	HGSOC	-	-	-	0
**2888**	missense	G245S	endometrioid	+	-	++	7.18
**2376**	missense	R248Q	HGSOC	+	+	+	2.1
**1951**	missense	R248W	HGSOC	+	+	+	0.21
**2893**	missense	R248W	endometrioid	+/-	+/-	+	0.5
**1878**	missense	R249W	endometrioid	+	+	+	1.6
**2096**	missense	R273H	endometrioid	+	+	++	22.16
**2534**	missense	R273H	HGSOC	+/-	+/-	++	2.34
**2813**	missense	R273H	HGSOC	+	-	-	0.07
**3153**	missense	R273H	mucinous	+	+	++	3
**3091**	missense	E286G	HGSOC	+	+	++	4.38
**641**	FS deletion	P75DEL	HGSOC	-	-	-	0
**3043**	FS deletion	K132DEL	undifferentiated	-	-	-	0
**369**	FS deletion	C242DEL	HGSOC	-	-	-	0
**104**	WT	-	mucinous	-	-	n.e.	n.e.
**874**	WT	-	mucinous	+/-	+/-	n.e.	n.e.
**1152**	WT	-	HGSOC	+	-	n.e.	n.e.
**257**	WT	-	HGSOC	+/-	-	n.e.	n.e.
**362**	WT	-	HGSOC	-	-	n.e.	n.e.
**654**	WT	-	HGSOC	-	-	n.e.	n.e.
**943**	WT	-	HGSOC	-	-	n.e.	n.e.
**2766**	WT	-	endometrioid	-	-	n.e.	n.e.
**3404**	WT	-	endometrioid	-	-	n.e.	n.e.

**1245**	WT	-	endometrioid	-	-	n.e.	n.e.
**2045**	WT	-	endometrioid	+	+/-	n.e.	n.e.
**1045**	WT	-	LGSOC	-	-	n.e.	n.e.
**1838**	WT	-	LGSOC	-	-	n.e.	n.e.
**1994**	WT	-	LGSOC	-	-	n.e.	n.e.
**766**	WT	-	LGSOC	+/-	+/-	n.e.	n.e.
**2748**	WT	-	undifferentiated	-	-	n.e.	n.e.
**1554**	WT	-	serous borderline	-	-	n.e.	n.e.
**2280**	WT	-	serous borderline	-	-	n.e.	n.e.
**2646**	WT	-	serous borderline	-	-	n.e.	n.e.
**2843**	WT	-	serous borderline	-	-	n.e.	n.e.
**2162**	WT	-	mucinous borderline	-	-	n.e.	n.e.
**1207**	WT	-	sero-mucinous borderline	-	-	n.e.	n.e.
**322**	missense	G248V	mucinous	+	+/-	n.e.	n.e.
**698**	missense	Y234C	mucinous	-	-	n.e.	n.e.
**2724**	missense	G244S	HGSOC	-	-	n.e.	n.e.
**3131**	missense	Y220C	HGSOC	+	+	n.e.	n.e.
**925**	missense	S215I	HGSOC	+	+	n.e.	n.e.
**970**	missense	Y234C	HGSOC	+	+/-	n.e.	n.e.
**1416**	missense	Y234C	HGSOC	+	+	n.e.	n.e.
**1144**	missense	Y220C	HGSOC	+	+/-	n.e.	n.e.
**2260**	missense	M237I	HGSOC	+	+	n.e.	n.e.
**2940**	missense	M237I	HGSOC	+	+	n.e.	n.e.
**3064**	missense	G266E	HGSOC	+	+	n.e.	n.e.
**3106**	missense	V216M	endometrioid	+	+/-	n.e.	n.e.
**3361**	missense	C135F	endometrioid	-	-	n.e.	n.e.
**1202**	missense	C238R	endometrioid	+	+	n.e.	n.e.
**1535**	missense	G244D	endometrioid	+	+	n.e.	n.e.
**2908**	missense	Y220C	endometrioid	+	+	n.e.	n.e.
**2875**	missense	S241A	endometrioid	**+**	+	n.e.	n.e.
**329**	missense	R248W	undifferentiated	+	+	n.e.	n.e.
**3342**	missense	R273L	undifferentiated	+	+	n.e.	n.e.
**2331**	missense	R249T	undifferentiated	+	-	n.e.	n.e.
**2624**	missense	Y220C	undifferentiated	+	+	n.e.	n.e.
**3519**	missense	R72H, R175H	undifferentiated	-	-	n.e.	n.e.
**1324**	missense	I254N	Carcinosarcoma	-	-	n.e.	n.e.
**3533**	FS deletion	227DEL	HGSOC	**+**	+/-	n.e.	n.e.
**3307**	FS deletion	261DEL	undifferentiated	**+**	**-**	n.e.	n.e.
**991**	nonsense	R196opa	HGSOC	**-**	**-**	n.e.	n.e.
**P53 aggregation positive**					**38/78**	**17/30**	**15/30**

“-“: negative. “+/-“: only some of the cells show a (weak) signal. “+”: positive. “++”: strong signal. “n.e.”: not evaluated. “HGSOC”: high-grade serous ovarian cancer. “LGSOC”: low-grade serous ovarian cancer. “WT”: wild-type. “FS deletion”: frameshift deletion

Supplementary Table 4: Association of total p53 protein expression determined by IF with p53 aggregation determined by co-IF, co-IP, and p53-Seprion-ELISA in OC patients.

		P53 aggregation detection method					
		**co-IF**		**co-IP**		**p53-Seprion-ELISA**	
		**positive**	**negative**	**positive**	**negative**	**positive**	**negative**
**p53 protein expression (IF)**	**positive**	38	8	17	4	15	6
	**negative**	0	32	0	9	0	9
		Cramer's V = 0.787, Fisher’s p < 0.001		Cramer's V = 0.675, Fisher’s p < 0.001		Cramer's V = 0.582, Fisher’s p < 0.001	

Supplementary Table 5: Association of OC subtype with p53 protein expression and p53 aggregation.

		**p53 protein expression**		**P53 aggregation detection method**					
		**IF**		**co-IF**		**co-IP**		**p53-Seprion-ELISA**	
		**positive**	**negative**	**positive**	**negative**	**positive**	**negative**	**positive**	**negative**
**Histology**	HGSOC	20	8	16	12	8	4	7	5
	LGSOC	1	5	1	5	0	2	0	2
	Endometrioid	11	5	10	6	5	1	4	2
	Mucinous	5	3	4	4	3	1	3	1
	Mucinous borderline	1	1	1	1	0	1	0	1
	Sero-mucinous borderline	2	1	2	1	0	2	0	2
	Serous borderline	1	5	1	5	1	1	1	1
	Undifferentiated	5	3	3	5	0	1	0	1
	Carcinosarcoma	0	1	0	1	0	0	0	0
		Cramer's V=0.408, Fisher's p=0.081		Cramer's V=0.334, Fisher's p=0.354		Cramer's V=0.596, Fisher's p=0.121		Cramer's V=0.516, Fisher's p=0.404	

3 References

1. Bairoch A. The Cellosaurus, a Cell-Line Knowledge Resource. Journal of biomolecular techniques : JBT. 2018;29(2):25-38.

2. de Andrade KC, Lee EE, Tookmanian EM, Kesserwan CA, Manfredi JJ, Hatton JN, et al. The TP53 Database: transition from the International Agency for Research on Cancer to the US National Cancer Institute. Cell Death & Differentiation. 2022;29(5):1071-3.
